# Subjects with Molecularly Defined Familial Hypercholesterolemia or Familial Defective apoB-100 Are Not Being Adequately Treated

**DOI:** 10.1371/journal.pone.0016721

**Published:** 2011-02-18

**Authors:** Trond P. Leren, Knut Erik Berge

**Affiliations:** Medical Genetics Laboratory, Department of Medical Genetics, Oslo University Hospital Rikshospitalet, Oslo, Norway; Finnish Institute of Occupational Health, Finland

## Abstract

**Objectives:**

To study whether subjects with a molecular genetic diagnosis of familial hypercholesterolemia (FH) or familial defective apoB-100 (FDB) are being adequately treated.

**Design:**

A questionnaire regarding medical history was sent to 2611 subjects who had been provided with a molecular genetic diagnosis of FH or FDB, and a blood sample was obtained for lipid measurements.

**Results:**

956 (36.6%) of the 2611 subjects participated. The mean age for starting lipid-lowering therapy was 33.4 (±12.1) years. Among those below 18 years of age, only 20.4% were on lipid-lowering drugs, whereas 89.1% of those aged 18 and above were on lipid-lowering drugs. The mean levels of total serum cholesterol and LDL-cholesterol were 5.7 (±1.5) mmol/l and 3.9 (±1.3) mmol/l, respectively. Among those who were on lipid-lowering drugs, 29.0% and 12.2% had levels of LDL cholesterol below 3.0 mmol/l and 2.6 mmol/l, respectively. Only 47.3% of the 956 subjects were considered as being adequately treated largely due to a failure to titrate their drug regimens. From the use of cholesterol-years score, lipid-lowering therapy must start before the age of 20 in order to prevent the subjects from contracting premature coronary heart disease.

**Conclusion:**

The majority of FH/FDB subjects are being diagnosed late in life and are not being adequately treated. In order to prevent them from contracting premature coronary heart disease, it is key that levels of LDL cholesterol are normalized from a young age and that sufficient doses of lipid-lowering drugs are being used.

## Introduction

Familial hypercholesterolemia (FH) is an autosomal dominant disorder caused by mutations in the low density lipoprotein (LDL) receptor gene [1]. As a result of defective cell-surface LDL receptors, clearance of LDL from plasma is reduced leading to increased plasma levels of LDL cholesterol and total cholesterol [1]. Those who are heterozygotes have values for total serum cholesterol in the range of 7 to 15 mmol/l, whereas those who are homozygotes have values for total serum cholesterol in the range of 20 to 25 mmol/l [1]. As a result of the increased cholesterol levels, FH subjects are at an increased risk of contracting premature coronary heart disease (CHD) [1], [2]. FH is one of the most common genetic disorders with a prevalence of heterozygotes of approximately 1/500 in most western countries [3], [4]. Thus, FH has a significant impact on public health and it has been estimated that FH heterozygotes constitute approximately 5% of those with premature CHD [5], [6].

However, efficient lipid-lowering therapy is available for FH heterozygotes which may normalize their levels of total serum cholesterol and LDL cholesterol [7]. The general experience, however, is that too few patients are being treated, and those who are being treated, are not being adequately treated [8]–[13]. Moreover, lipid-lowering therapy generally starts late in life at a time when advanced atherosclerosis has already developed [13]. It is believed that a major cause for these treatment failures is difficulties in diagnosing FH by the use of the clinical diagnostic criteria consisting of autosomal dominant hypercholesterolemia and presence of premature CHD and xanthomas [1], [14]–[16]. The sensitivity of these criteria is particularly low in children/adolescents who do not have manifest CHD nor xanthomas. In contrast to the relatively vague clinical criteria, a specific diagnosis can be obtained by identifying a mutation in the LDL receptor gene by molecular genetic methods [1], [10], [16].

Two genocopies of FH have been identified. One is familial defective apoB-100 (FDB) which is caused by mutation R3500Q in the apoB-100 gene [17], and the other is FH3 which is caused by gain-of-function mutations in the proprotein convertase subtilisin/kexin type 9 (PCSK9) gene [18]. Among subjects provided with a molecular genetic diagnosis of autosomal dominant hypercholesterolemia in Norway, 95% are FH heterozygotes, 3% are FDB heterozygotes and 2% are FH3 heterozygotes (unpublished data).

To study whether a molecular genetic diagnosis of autosomal dominant hypercholesterolemia secures that the subjects become adequately treated, we have performed a questionnaire-based study and performed lipid measurements among subjects who have been provided with a molecular genetic diagnosis of FH or FDB during a 10-year period in Norway.

## Materials and Methods

### Subjects

During the period 1998–2008, 2611 subjects have been provided with a molecular genetic diagnosis of FH or FDB in our laboratory. This had been done by the use of various mutations detection methods to screen for mutations in the LDL receptor gene (NG_009060) and apoB-100 gene (NG_011793), respectively. The subjects were invited to participate in a follow-up study to have their current therapies evaluated. Thus, the main aim of the study was to secure that subjects who had been diagnosed with FH or FDB, were being adequately treated. In order to participate, the subjects would have to answer a questionnaire for assessment of their risk profile and to have a non-fasting blood sample drawn for lipid analyses. The blood samples were sent to Department of Medical Biochemistry, Oslo University Hospital Rikshospitalet for analysis, and the report was sent to us. Values for LDL cholesterol were obtained by direct measurement and not calculated according to the formula of Friedewald et al. [19]. Thus, measurements of values for LDL cholesterol were not dependent on fasting blood samples. Because, the goal of this initiative was to secure that patients were being adequately treated, it did not need approval of an ethics committee as confirmed by a statement of August 30, 2006 from Regional Committee for Research Ethics in Southern Norway. Thus, the ethics committee specifically waived the need to collect written informed consent.

### Evaluation of the adequacy of the current lipid-lowering therapy

Evaluation of the adequacy of the current lipid-lowering therapy was based upon assessment of the subject's global risk for CHD from medical and family histories obtained at the time of genetic testing and from information in the questionnaire. Several factors were considered for this evaluation such as age of the subject, family history, lipid levels before lipid-lowering therapy was started, age for start of lipid-lowering therapy, number of years on lipid-lowering therapy, lipid profile on lipid-lowering therapy, presence of manifest CHD such as myocardial infarction or angina pectoris and the presence of additional risk factors such as smoking and hypertension. Based upon the subject's risk profile which was established by the two authors who are well experienced with genetic lipid disorders, the current lipid profile was evaluated and the lipid-lowering therapy was classified as being adequate or not. No defined algorithm was being used for this classification, as there is no validated algorithm that considers all relevant parameters for assessing the global risk for CHD in FH/FDB heterozygotes. However, with respect to the lipid profile the values for total serum cholesterol and LDL cholesterol should generally be below 5 mmol/l and 3 mmol/l, respectively for the therapy to be considered adequate.

## Results

Of the 2611 subjects with a molecular genetic diagnosis of FH or FDB who had been invited to participate, 956 (36.6%) actually answered the questionnaire and had a blood sample drawn for lipid measurements. Descriptive statistics of the participants are given in Table 1. There were 534 females and 422 males and there was a clear tendency towards relative more females in the higher age groups. This probably reflects that females develop CHD later in life than males. The mean age of the subjects was 44.3 (±17.0) years. The youngest was 5 and the oldest was 77 years old. Molecular genetic testing with respect to FH or FDB had been performed at a mean age of 39.8 (±17.3) years, and 35 subjects were heterozygous for the R3500Q mutation in the apoB-100 gene and 921 were heterozygous for one of 88 different mutations in the LDL receptor gene. 266 (27.8%) subjects were index patients and 690 (72.2%) had been diagnosed by cascade genetic screening.

**Table 1:** Characteristics of subjects with FH or FDB of different age groups who participated in the follow-up study to evaluate their treatment status.

Age groups								
	All	5–14	15–24	25–34	35–44	45–54	55–64	>65
N (males/females)	422/534	29/21	54/54	37/64	94/100	82/117	158/226	50/169
Age (years)	44.3 (±17.0)	12.2 (±2.1)	18.7 (±2.9)	29.9 (±3.1)	39.8 (±2.8)	49.2 (±2.8)	54.1 (±5.8)	69.1 (±3.3)
Age of moleculargenetic testing (years)	39.8 (±17.3)	8.8 (±2.9)	13.7 (±3.7)	25.4 (±4.0)	35.1 (±3.5)	44.3 (±3.8)	49.4 (±6.6)	65.4 (±4.0)
Index patients (%)	27.8	12.0	10.2	29.7	35.1	34.5	33.6	18.5
Age for measurementof total serum cholesterolwithout lipid-loweringtherapy (years)	27.4 (±13.3)	6.5 (±3.1)	8.9 (±4.8)	18.8 (±6.0)	24.5 (±7.3)	29.4 (±8.8)	33.0 (±10.0)	41.0 (±12.2)
Value for total serumcholesterol withoutlipid-lowering therapy (mmol/l)	10.5 (±2.5)	8.2 (±1.5)	8.5 (±1.6)	9.1 (±1.8)	10.1 (±2.5)	11.0 (±2.4)	11.1 (±2.4)	11.9 (±2.4)
Age for starting lipid-loweringdrugs (years)	33.4 (±12.1)	10.5 (±1.1)	15.4 (±3.1)	22.0 (±5.1)	29.5 (±6.5)	33.7 (±8.3)	37.4 (±9.7)	45.3 (±11.7)
Manifest CHD# (%)	16.1	0	0	1.0	9.8	14.7	20.2	42.7
Current smoker (%)	13.5	0	9.3	9.0	13.9	20.8	19.3	7.6
Hypertension (%)	12.1	0	1.9	3.2	5.3	11.2	15.2	35.1

# Myocardial infarction, angina pectoris, coronary artery bypass graft or percutaneous coronary intervention.

**Figure 1:**
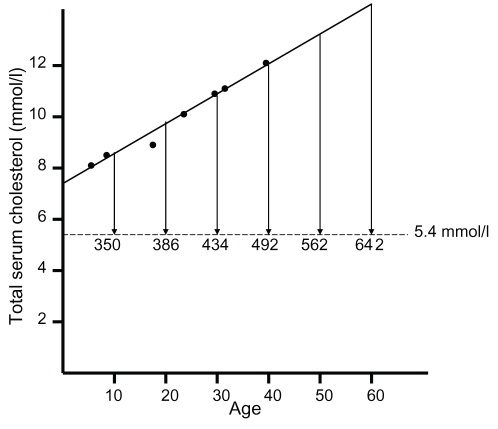
Cholesterol-years score in FH/FDB heterozygotes. Levels of total serum cholesterol in FH/FDB heterozygotes in different age groups before lipid-lowering therapy is started are plotted against age of the subjects (data from Table 1). Linear regression (y = 0.111x +7.408) has been used to generate the relationship between levels of total serum cholesterol and age from birth until the age of 60. Cholesterol-years scores for subjects who are put on lipid-lowering therapy at different decades and achieved a total serum cholesterol level of 5.4 mmol/l, are indicated.

The mean age for the first measurement of total serum cholesterol without lipid-lowering therapy was 27.4 (±13.3) years, and the reported value for total serum cholesterol was 10.5 (±2.5) mmol/l (Table 1). The relationship between age and levels of total serum cholesterol before lipid-lowering therapy was started, is shown in Fig. 1. 84.8% of the subjects had used lipid-lowering drugs, and the mean age for starting lipid-lowering therapy was 33.4 (±12.1) years (Table 1). 16.1% reported that they had manifest CHD, 13.5% were current smokers and 12.1% had hypertension.

At the follow-up, 82.3% were on lipid-lowering drugs (Table 2). However, among those below age 18, only 20.4% were on lipid-lowering drugs, whereas 89.1% of those aged 18 and above were on lipid-lowering drugs. Among the subjects aged 30 and below or 40 and below, 45.3% and 62.9%, respectively were on lipid-lowering drugs.

**Table 2:** Lipid-lowering therapy in subjects with FH or FDB of different age groups at the follow-up.

Age groups								
	All	5–14	15–24	25–34	35–44	45–54	55–64	>65
Number on lipid-lowering therapy (%)	82.3	16.0	46.6	78.8	85.8	96.4	95.8	95.8
Total serumcholesterol (mmol/l)	5.7 (±1.5)	6.7 (±1.3)	6.0 (±1.7)	5.9 (±1.8)	5.6 (±1.5)	5.5 (±1.4)	5.6 (±1.4)	5.6 (±1.4)
HDL cholesterol (mmol/l)	1.4 (±0.4)	1.3 (±0.3)	1.3 (±0.3)	1.4 (±0.4)	1.3 (±0.4)	1.4 (±0.4)	1.4 (±0.4)	1.5 (±0.5)
Triglycerides (mmol/l)	1.0 (±0.6)	0.9 (±0.4)	1.0 (±0.7)	1.0 (±0.7)	1.0 (±0.6)	1.1 (±0.6)	1.1 (±0.6)	1.1 (±0.6)
LDL cholesterol (mmol/l)	3.9 (±1.3)	4.8 (±1.2)	4.1 (±1.4)	4.0 (±1.6)	3.8 (±1.3)	3.7 (±1.2)	3.7 (±1.2)	3.7 (±1.3)
Reduction in levels oftotal serum cholesterol (%)	45.7	18.3	29.4	35.2	44.6	50.0	49.5	52.9
Achieved levels of LDLcholesterol <3 mmol/l (%)	29.0	0	27.1	33.3	31.9	27.8	29.6	26.3
Therapy consideredadequate (%)	52.7	36.0	56.5	52.5	47.4	57.0	54.4	59.7

The levels of total serum cholesterol and LDL cholesterol measured at the follow-up for the 956 subjects, were 5.7 (±1.5) mmol/l and 3.9 (±1.3) mmol/l, respectively (Table 2). Thus, compared to the reported levels of total serum cholesterol before lipid-lowering therapy were started, a reduction in the levels of total serum cholesterol of 45.7% had been achieved. The mean value for total serum cholesterol in the 772 subjects who were on lipid-lowering therapy, was 5.4 (±1.2) mmol/l (LDL cholesterol: 3.6 (±1.2) mmol/l), and these subjects had achieved a reduction in levels of total serum cholesterol of 49.5%. Among the 772 subjects who were on lipid-lowering therapy, 34.9% were on atorvastatin monotherapy with a mean dosis of 53 mg, and 29.3% were on simvastatin monotherapy with a mean dosis of 43 mg. 22.5% were on combination therapy with atorvastatin and ezetimibe, 4.8% were on combination therapy with simvastatin and ezetimibe and 3.7% were on combinatation therapy with three or more lipid-lowering drugs.

Among those who were on lipid-lowering therapy, only 29.0% had an LDL cholesterol value below 3.0 mmol/l, and only 12.2% had an LDL cholesterol value below 2.6 mmol/l. Among all 956 subjects, 52.7% were considered as being inadequately treated (Table 2). The levels for total serum cholesterol and LDL cholesterol among those whose lipid-lowering therapies were considered as being inadequate, were 6.6 (±1.4) mmol/l and 4.7 (±1.3) mmol/l, respectively. These levels were significantly higher than the corresponding levels of 4.9 (±1.1) mmol/l (p<0.0001) and 3.1 (±0.9) mmol/l (p<0.0001), respectively in those whose therapy was considered as being adequate.

There was a wide variation regarding the age both for diagnosis and for the start of lipid-lowering therapy (Table 1). Some had been diagnosed and put on lipid-lowering therapy in their first or second decades, whereas others had not been diagnosed and put on lipid-lowering therapy until their sixth or seventh decades. The later in life lipid-lowering therapy starts, and the higher the total serum cholesterol level, the more atherosclerosis is present [20]. The cholesterol-years score can be used as a measurement of the atherosclerosis burden [21]. To determine the cholesterol-years score, the value for total serum cholesterol is multiplied by the age of the subject to generate the “area under the curve” in Fig. 1.

If the mean value for total serum cholesterol in the 772 subjects who were on lipid-lowering therapy at the follow-up of 5.4 (±1.2) mmol/l, is being used as a fixed value for total serum cholesterol on lipid-lowering therapy, the data in Fig. 1 can be used to calculate the cholesterol-years score for subjects who start lipid-lowering therapy at different ages until the seventh decade. Whereas, a non-FH/FDB subject with a value for total serum cholesterol of 5.4 mmol/l until the age of 60 will have a cholesterol-years score of 324 mmol years/l, an FH/FDB subject who does not start lipid-lowering therapy before the age of 60, will have a cholesterol-years score of 642 mmol years/l (Fig. 1). However, an FH/FDB subject who starts lipid-lowering therapy at the age of 10 and achieves a total serum cholesterol level of 5.4 mmol/l, will have a cholesterol-years score of 350 mmol years/l at the age of 60 (Fig. 1). For subjects who starts lipid-lowering therapy at the age of 10 or 20 years, treatment values for total serum cholesterol must be 4.9 mmol/l or 3.9 mmol/l, respectively in order to achieve a cholesterol-years score of 324 mmol years/l at the age of 60.

## Discussion

In this study we have determined the treatment status of FH/FDB patients provided with a molecular genetic diagnosis during the period 1998–2008 in Norway. The main finding was that even though the subjects had obtained a specific molecular genetic diagnosis, only 47.3% were considered as being adequately treated. This finding reflects that submaximal doses of lipid-lowering drugs were used with mean doses of atorvastatin and simvastatin monotherapies of 53 mg and 43 mg, respectively. Thus, there is a need to educate physicians in order to improve the lipid-lowering therapy in FH/FDB patients. Moreover, our study has shown that lipid-lowering therapy had been started late in life at a mean age of 33.4 (±12.1) years. This partly reflects that the mean age for the first measurement of total serum cholesterol was 27.4 (±13.3) years and that the mean age for the molecular genetic diagnosis was 39.8 (±17.3) years. Thus, there is a need to diagnose these subjects earlier in life.

However, some caution should be exerted when interpreting our findings since only 36.6% of those who were invited to participate, actually did participate. The participants could therefore in theory represent a selected group of patients who could be particularly well or particularly poorly treated. Moreover, because information regarding medical history was obtained from the patients themselves through a questionnaire, and not extracted from medical files, some uncertainties may exist regarding the quality of this information.

Among those aged 18 and above, 89.1% were on lipid-lowering drugs. For comparison 96% of adult Dutch FH patients were on lipid-lowering drugs [22]. This minor difference may partly reflect that the Dutch patients were treated at specialized out-patient clinics, whereas the majority of the Norwegian patients were being treated by general practitioners. Among the Norwegian patients who were on lipid-lowering drugs, 29.0% and 12.2% had levels of LDL cholesterol below 3.0 mmol/l and 2.6 mmol/l, respectively. Thus, the majority of Norwegian FH/FDB subjects are being diagnosed late in life and are not being adequately treated. 16% of the subjects in our study had a history of CHD which is similar to that of 17% found among Dutch FH heterozygotes [22].

The American Academy of Pediatrics has recommended that screening for FH should be performed at an age of 2–10 years, and that lipid-lowering therapy may start from the age of 8 [23]. These recommendations reflect that the atherosclerotic lesions are largely reversible before the age of 10 in contrast to the calcified lesions seen in untreated middle-aged FH/FDB subjects. Moreover, it has been well documented that lipid-lowering therapy from late childhood/adolescence is effective, safe and well tolerated [24].

The cholesterol-years score in Fig. 1 illustrates the importance of starting lipid-lowering therapy at a young age. This score has been found to be a predictor of aortic stenosis [25], [26] and is correlated with the severity of atherosclerosis in carotid arteries in FH heterozygotes [27], [28]. It also correlates well with the severity of calcific atherosclerosis and angina pectoris in FH homozygotes with an apparent threshold for calcific atherosclerosis at a cholesterol-years score of 182 mmol l/years [21]. If lipid-lowering therapy starts at the age of 10, and a realistic treatment value for total serum cholesterol of 4.9 mmol/l is achieved, the cholesterol-years score until the age of 60 is 324 mmol year/l. This score is identical to that of a non-FH/FDB subject who has had a total serum cholesterol level of 5.4 mmol/l during the same periode. For comparison, the median levels of total serum cholesterol in the Nordic countries for subjects in their fourth, fifth and sixth decades are 4.8 mmol/l, 5.2 mmol/l and 5.6 mmol/l, respectively [www.furst.no/norip/]. If lipid-lowering therapy starts at the age of 20, a total serum cholesterol value of 3.9 mmol/l is required to achieve a cholesterol-years score of 324 mmol year/l at the age of 60. Such a cholesterol value will probably only be achievable in a minority of FH/FDB patients. If lipid-lowering therapy starts after the second decade, it is not realistic to achieve sufficiently low levels of total serum cholesterol and LDL cholesterol to achieve a risk of CHD at the age of 60 similar to that of a non-FH/FDB subject. Thus, for subjects diagnosed after the age of 20, maximum tolerated doses of combination therapies should be prescribed in order to reduce the risk of CHD.

The question then is how secure that patients with FH/FDB are being diagnosed early in life. Several strategies have been discussed, including population-based cholesterol screening of children which has not been recommended by any pediatric organizations [29]. Because lipid measurements do not have the required sensitivity and specificity and because clinical characteristics such as xanthomas and premature CHD do not occur in children, the diagnosis should preferentially be based on molecular genetic testing [16]. Our recommendation which is in line with the NICE guidelines [30] is that cholesterol screening is made common in young adults in order to select index patients for molecular genetic screening. Once the underlying mutation has been identified, cascade genetic screening, which is the most cost-effective strategy to diagnose FH/FDB patients [31]-[33], is being used to diagnose children and other family members. However, our study clearly illustrates the need for educating physicians in order to improve the lipid-lowering therapy even in FH/FDB patients with a molecular genetic diagnosis.

## References

[1] Goldstein JL, Hobbs HH, Brown MS, Scriver CR, Baudet AL, Sly WS, Valle D (2001). Familial hypercholesterolemia.. The metabolic basis of inherited disease.

[2] Slack J (1969). Risks of ischaemic heart disease in familial hyperlipoproteinaemic states.. Lancet.

[3] Heiberg A, Berg K (1976). The inheritance of hyperlipoproteinemia with xanthomatosis. A study of 132 kindreds.. Clin Genet.

[4] Slack J, Paoletti R, Gotto AM (1979). Inheritance of familial hypercholesterolemia.. Atheroscl Rev.

[5] Williams RR, Hopkins PN, Hunt SC, Wu LL, Hasstedt SJ (1990). Population-based frequency of dyslipidemia syndromes in coronary-prone families in Utah.. Arch Intern Med.

[6] Koivisto UM, Hämäläinen L, Taskinen MR, Kettunen K, Kontula K (1993). Prevalence of familial hypercholesterolemia among young north Karelian patients with coronary heart disease: a study based on diagnosis by polymerase chain reaction.. J Lipid Res.

[7] Huigen R, Vissers MN, Defesche JC, Lansberg PJ, Kastelein JJ (2008). Familial hypercholesterolemia: current treatment and advances in management.. Expert Rev Cardiovasc.

[8] Williams RR, Hamilton-Craig I, Kostner GM, Hegele RA, Hayden MR, Berg K, Boulyjenkov V, Christen Y (1996). MED PED: An integrated genetic strategy for preventing early deaths.. Genetic approaches to noncommunicable diseases.

[9] Neil HAW, Hammond T, Huxley R, Matthews DR, Humphries SE (2000). Extent of underdiagnosis of familial hypercholesterolemia in routine practice: prospective registry study.. BMJ.

[10] Maarle MCV, Stouthard MEA, Mheen JMVD, Klazinga NS, Bonsel GJ (2002). Follow up after a family based screening programme for familial hypercholesterolemia: is screening alone enough?. BMJ.

[11] Umans-Eckenhausen MAW, Defesche JC, Sijbrands EJG, Scheerder RLJM, Kastelein JJP (2001). Review of the first 5 years of screening for familial hypercholesterolemia in the Netherlands.. Lancet.

[12] Umans-Eckenhausen MAW, Defesche JC, van Dam MJ, Kastelein JJP (2003). Long-term compliance with lipid-lowering medication after genetic screening for familial hypercholesterolemia.. Am Med Ass.

[13] Leren TP, Finborud TH, Manshaus TE, Ose L, Berge KE (2008). Diagnosis of familial hypercholesterolemia in general practice using clinical diagnostic criteria or genetic testing as part of cascade genetic screening.. Community Genet.

[14] Scientific Steering Committee on behalf of the Simon Broome Register Group (1991). Risk of fatal coronary heart disease in familial hypercholesterolemia.. BMJ.

[15] Defesche J, Betteridge DJ (2000). Familial hypercholesterolemia.. Lipids and vascular disease.

[16] Leren TP, Berge KE (2009). Comparison of clinical and molecular genetic criteria for diagnosing familial hypercholesterolemia.. Clin Lipidol.

[17] Soria LF, Ludwig EH, Clarke HRG, Vega GL, Grundy SM (1989). Association between a specific apolipoprotein B mutation and familial defective apolipoprotein B-100.. Proc Natl Acad Sci USA.

[18] Mousavi SA, Berge KE, Leren TP (2009). The unique role of proprotein convertase subtilisin/kexin 9 in cholesterol homeostasis.. J Intern Med.

[19] Friedewald WT, Levy RI, Fredrickson DS (1972). Estimation of the concentration of low density lipoprotein cholesterol in plasma, without use of preparative ultracentrifuge.. Clin Chem.

[20] Law MR, Wald NJ (1995). An ecological study of serum cholesterol and ischaemic heart disease between 1950 and 1990.. Eur J Clin Nutr.

[21] Hoeg J, Feuerstein IM, Tucker EE (1994). Detection and quantitation of calcific atherosclerosis by ultrafast computed tomography in children and young adults with homozygous familial hypercholesterolemia.. Arterioscler Thromb.

[22] Pijlman AH, Huigen R, Verhagen SN, Imholz BP, Liem AH (2010). Evaluation of cholesterol lowering treatment of patients with familial hypercholesterolemia: A large cross-sectional study in The Netherlands.. Atherosclerosis.

[23] Daniels SR, Greer FR (2008). Lipid screening and cardiovascular health in childhood.. Pediatrics.

[24] Vuorio A, Kuoppala J, Kovanen PT, Humphries SE, Strandberg T (2010). Statins for children with familial hypercholesterolemia.. Cochrane Database Syst Rev.

[25] Nozue T, Kawashiri M, Higashikata T, Nohara A, Inazu A (2006). Cholesterol-years score is associated with development of senile degenerative aortic stenosis in heterozygous familial hypercholesterolemia.. J Atheroscler Thromb.

[26] Rallidis L, Naoumova RP, Thompson GR, Nihoyannopoulos P (1998). Extent and severity of atherosclerotic involvement of the aortic valve and root in familial hypercholesterolemia.. Heart.

[27] Tonstad S, Joakimsen O, Stensland-Bugge E, Ose L, Bønaa KH (1998). Carotid intima-media thickness and plaque in patients with familial hypercholesterolaemia mutations and control subjects.. Eur J Clin Invest.

[28] Sidhu PS, Naoumova RP, Maher VM, MacSweeney JE, Neuwirth CK (1996). The extracranial carotid artery in familial hypercholesterolaemia: relationship of intimal-medial thichness and plaque morphology with plasma lipid and coronary heart disease.. J Cardiovasc Risk.

[29] US preventive task force (2007). Screening for lipid disorders in children: US preventive task force statement.. Pediatrics.

[30] Wierzbicki AS, Humphries SE, Minhas R (2008). Familial hypercholesterolaemia: summary of NICE guidance.. BMJ.

[31] Marks D, Wonderling D, Thorogood M, Lambert H, Humphries SE (2000). Screening for hypercholesterolaemia versus case finding for familial hypercholesterolaemia: a systematic review and cost-effectiveness analysis.. Health Technol Assess.

[32] Wonderling D, Umans-Eckenhausen MA, Marks D, Defesche JC, Kastelein JJ (2004). Cost-effectiveness analysis of the genetic screening program for familial hypercholesterolemia in The Netherlands.. Semin Vasc Med.

[33] Leren TP (2004). Cascade genetic screening for familial hypercholesterolemia.. Clin Genet.

